# Neuromyelitis Optica Spectrum Disorder: A Case Report

**DOI:** 10.31729/jnma.8748

**Published:** 2024-09-30

**Authors:** Prakriti Karki, Sagar Pahari, Nayan Neupane, Kushal Poudel, Nehal Asharaf

**Affiliations:** 1 Tairunnessa Memorial Medical College and Hospital, Gazipur, Bangladesh; 2 Tribhuvan University Teaching Hospital, Maharajgunj, Kathmandu, Nepal; 3 Nepal Medical College, Jorpati, Kathmandu, Nepal; 4 Nepalgunj Medical College, Nepalgunj, Banke, Nepal; 5 Bir Hospital, Mahaboudha, Kathmandu, Nepal

**Keywords:** *aquaporin 4*, *devic disease*, *neuromyelitis optica spectrum disorder*

## Abstract

Neuromyelitis optica spectrum disorder also known as Devic's disease is an autoimmune condition where the body produces antibodies against Aquaporin-4 in the astrocytes. This affects the brain and spinal cord leading to numerous manifestations like paralysis, transverse myelitis, and optic neuritis. MRI and aquaporin-4 antibody (AQP4-IgG) are crucial for its diagnosis. Treatment of the acute stage involves plasma exchange and intravenous steroids. Steroids and immune modulators can do long-term management. This case highlights a 40-year-old woman who manifested a wide range of neurological symptoms, including paralysis and eyesight loss. She had blood testing, radiology, and a clinical evaluation to diagnose her condition. Neuromyelitis optica spectrum disorder was diagnosed in light of the investigations. This instance emphasizes the need for careful examination and consideration of any paralysis. To prevent patients from experiencing psychological, financial, or physical challenges, treating physicians should be vigilant for any findings related to conditions like neuromyelitis optica spectrum disorder.

## INTRODUCTION

Neuromyelitis Optica Spectrum Disorder (NMOSD) is an autoimmune disease that causes blindness and paralysis by attacking the aquaporin-4 (AQP4) water channel of astrocytes in the optic nerves and spinal cord.^1^ With an estimated prevalence of 0.5 to 10 cases per 100,000 people NMOSD is an uncommon condition.^2^ The immune system's induced secondary demyelination and astrocyte destruction cause acute episodes, which can cause paralysis, transverse myelitis, other neuropathological diseases, or even death. Significant vision impairment from unilateral or bilateral optic neuritis, as well as unexplained nausea, vomiting, or hiccups from region postrema syndrome, are further distinguishing characteristics.^3^ Hereby we report a case of 40 years female Diagnosed with NMOSD.

## CASE REPORT

A 40 year female had progressive decreased vision in her left eye over 10 days which later caused complete loss of left vision. This was later followed by a reduction of vision of right eye after a few days. MRI (Magnetic Resonance Imaging) of the brain revealed no abnormality and after a few days, the right eye's vision recovered gradually. In between she had no other symptoms except of impaired left-sided vision. She was advised for ophthalmologist consultation by her treating physician which was not followed. After 4 months she had progressive weakness in her right leg which began as a tingling sensation of sole. It was also associated with weakness and dragging of the foot which required support. There was numbness and tingling in the left leg but no loss of power. These events were accompanied by constipation and a bandlike sensation over the T9 and T10 dermatome levels. Over a few months, her power improved and she could move her lower limbs. After 12 months she started to develop urinary incontinence and extreme weakness of bilateral lower limbs. This time weakness was so severe that she could not stand without support. For this, she was managed with Inj dexamethasone 8 mg IV 8 hourly during her stay in the hospital which relieved her symptoms and her urinary incontinence improved slightly which led her to walk independently.

**Figure 1:**
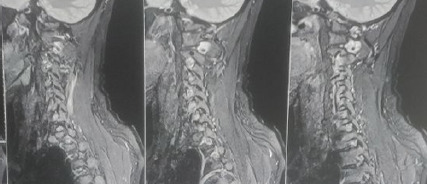
MRI showing high signaling intensity area involving the cervical spinal cord extending from cervicomedullary junction to C7 vertebrae

After 1 month she developed severe weakness of her lower limbs, this time it which was associated with spontaneous contraction and numbness. She also had cramps in her upper limbs which were more severe in her right than in her left, this was accompanied by weakness that was so severe that she could not hold anything with her right hand. On examination there was RAPD (Relative Afferent Pupil Defect) in the left eye, power was found to be 4/5 in the right upper limb, and Hoffman's sign was positive in the left upper limbs. Lower limb examinations showed power to be 0/5 on the right and 1/5 on the left, planter reflex was extensor in bilateral limbs. Signs of meningeal irritations were negative on examination and she was afebrile during her entire hospital stay. MRI of the cervical spine revealed a mildly enhancing long T2 segment with a high signaling intensity area involving the cervical spinal cord extending from the cervicomedullary junction to C7 vertebrae, suggesting longitudinally extensive transverse myelitis (Figure 1). Antibody against Aquaporin-4 (AQP4) was found to be positive. Other investigations including Myelin oligodendrocyte glycoprotein (MOG), Angiotensinconverting enzyme(ACE),Anti Nuclear Antibody(ANA) were not significant during the hospital stay. Tests for AQP4, MOG, and ACE were performed in a lab outside of the hospital as they are not commonly performed and are not available in government centers in Nepal. A single (Cerebro Spinal Fluid) study during the initial hospital stay revealed TLC-3/mm3, Monomorph-0%, polymorph-0%, and sugar -3mmol/L within the normal range which excludes any probability of meningitis. She was managed with plasma exchange and steroids in the hospital while being discharged on oral steroids and muscle relaxants. After a few weeks, her symptoms got better and she was discharged with proper counseling. During her hospital stay, she was consulted by the physiotherapist and was advised to follow exercises as they would improve her muscle tone and be helpful for her recovery process. On follow-up after 2 weeks her motor function improved and she responded well to her treatment as suggested. She was advised for immune suppressive therapy but was denied because of the need for prolonged use and poor financial condition.

## DISCUSSION

NMOSD is an autoimmune condition that targets the astrocyte aquaporin-4 (AQP4).^1^ AQP4 is the most extensively expressed water channel. AQP4 is found particularly found in the foot processes of astrocytes at the blood-brain barrier. Additionally, AQP4 is found in the kidney's collecting ducts, the stomach's parietal cells, the airways, the secretory glands, and the skeletal muscle. However, because the brain lacks local complement inhibitors, these organs are comparatively protected.^3^

Acute disseminated encephalomyelitis, transverse myelitis, optic neuritis, brainstem pathology frequently affecting cerebellar peduncles, involvement of cranial nerves, and, less frequently, brainstem encephalitis, encephalitis mimicking small vessel CNS vasculitis, and cortical disease with seizures are among the conditions that affected patients may develop.^4^ The main clinical features include optic neuritis, acute myelitis, area postrema syndrome (hiccups, nausea, and/or vomiting), acute brainstem syndrome, narcolepsy, or acute diencephalic clinical syndrome with a typical diencephalic MRI lesion, as well as symptomatic cerebral syndrome with a typical NMOSD brain lesion.^5^

Patients with NMOSD who have AQP4-IgG must meet certain fundamental clinical requirements, which include the presence of clinical syndromes or MRI abnormalities about the optic nerve, spinal cord, region postrema, other brainstem, diencephalic, or cerebral presentations. For the diagnosis of NMOSD in the absence of AQP4-IgG or in situations where serologic testing is not accessible, more exacting clinical criteria together with additional neuroimaging findings are needed.^6^ NMOSD risk is higher in patients with AITD (autoimmune thyroid disease), SLE (systemic lupus erythematosus), and SS (systemic sclerosis). It is discovered that the pathophysiology of NMOSD coexisting with AITD, SLE, and SS may include biological processes connected to MHC class I and the interferon-gamma-mediated signaling system.^7^

IVMP (IV methylprednisolone) and PLEX (Plasma exchange) are used in acute conditions. IVIgG(IV immunoglobulin G) is used as a treatment if there is a poor response. Trials are being conducted on Bevacizumab and Ubilituximab for acute treatment. Trials are being conducted on Azathioprine, Mycophenolate mofetil, Rituximab, and Tocilizumab to determine their long-term efficacy.^8^

This case offers important new information about NMOSD. The comprehensive assessment, including diagnostic imaging and blood tests, contributes to a well-supported diagnosis. Although this case gives successful management of acute conditions but doesn't get into long-term management. Early diagnosis and management of NMSOD are crucial and help to prevent further deterioration. To effectively manage, one must have a complete awareness of this condition and the most recent therapy approaches but financial issues lie for the newer drugs. The clinical findings in this instance contribute to our knowledge of NMOSD and highlight the value of early, proactive care in cases like this.

## References

[1] Holroyd KB, Manzano GS, Levy M (2020). Update on neuromyelitis optica spectrum disorder.. Curr Opin Ophthalmol..

[2] Fiala C, Rotstein D, Pasic MD (2022). Pathobiology, Diagnosis, and Current Biomarkers in Neuromyelitis Optica Spectrum Disorders.. J Appl Lab Med..

[3] Huda S, Whittam D, Bhojak M, Chamberlain J, Noonan C, Jacob A (2019). Neuromyelitis optica spectrum disorders.. Clin Med (Lond)..

[4] Asseyer S, Cooper G, Paul F (2020). Pain in NMOSD and MOGAD: A Systematic Literature Review of Pathophysiology, Symptoms, and Current Treatment Strategies..

[5] Höftberger R, Lassmann H (2018). Inflammatory demyelinating diseases of the central nervous system.. Handbook of Clinical Neurology. Elsevier B.V..

[6] Wingerchuk DM, Banwell B, Bennett JL, Cabre P, Carroll W, Chitnis T (2015). VIEWS & REVIEWS International consensus diagnostic criteria for neuromyelitis optica spectrum disorders..

[7] Wang X, Shi Z, Zhao Z, Chen H, Lang Y, Kong L (2022). The causal relationship between neuromyelitis optica spectrum disorder and other autoimmune diseases.. Front Immunol..

[8] Carnero Contentti E, Correale J (2021). Neuromyelitis optica spectrum disorders: from pathophysiology to therapeutic strategies.. Vol. 18, Journal of Neuroinflammation. BioMed Central Ltd.

